# The Impact of Microplastics in Food and Drugs on Human Health: A Review of the MENA Region

**DOI:** 10.3390/ijerph22030380

**Published:** 2025-03-05

**Authors:** Noha Alziny, Fadya M. Elgarhy, Ayan Musa Mohamed, Hager Yehia Shalaby, Noran Nounou, Omnia Soliman, Anwar Abdelnaser

**Affiliations:** Institute of Global Public Health and Human Ecology, School of Sciences and Engineering, The American University in Cairo, Cairo 11835, Egypt; nohaalziny@aucegypt.edu (N.A.); fadya_elgarhy@aucegypt.edu (F.M.E.); ayan@aucegypt.edu (A.M.M.); hagershalaby@aucegypt.edu (H.Y.S.); noran.nounou@aucegypt.edu (N.N.); omniasoliman@aucegypt.edu (O.S.)

**Keywords:** microplastic (MP), MENA, Egypt, food system, human health, public health, neuroinflammatory disorders, neurodegenerative diseases

## Abstract

Microplastics (MPs), defined as plastic particles smaller than 5 mm, have emerged as a global environmental and public health crisis, infiltrating air, water, soil, and food systems worldwide. MPs originate from the breakdown of larger plastic debris, single-use plastics, and industrial processes, entering food. Emerging evidence underscores the ability of MPs to cross biological barriers, including the blood–brain barrier, triggering neuroinflammatory responses and contributing to neurodegenerative diseases such as Alzheimer’s and Parkinson’s. Polystyrene (PS), a common type of MP, activates microglial cells, releasing pro-inflammatory cytokines like tumor necrosis factor (TNF-α) and interleukins, which increase neuronal damage. MPs have also been linked to cardiovascular diseases, with studies detecting polyethylene (PE) and polyvinyl chloride (PVC) in carotid artery plaques, increasing the risk of myocardial infarction and stroke. Furthermore, MPs disrupt endocrine function, alter lipid metabolism, and induce gut microbiome imbalances, posing multifaceted health risks. In the MENA region, MP pollution is particularly severe, with the Mediterranean Sea receiving an estimated 570,000 tons of plastic annually, equivalent to 33,800 plastic bottles per minute. Studies in Egypt, Lebanon, and Tunisia document high MP concentrations in marine ecosystems, with herbivorous fish like *Siganus rivulatus* containing over 1000 MPs per individual due to the ingestion of contaminated seaweed. Despite these findings, public awareness and regulatory frameworks remain inadequate, with only 24% of Egyptians demonstrating sufficient knowledge of safe plastic use. This review emphasizes the urgent need for region-specific research, policy interventions, and public awareness campaigns to address MP pollution. Recommendations include sustainable waste management practices, the promotion of biodegradable alternatives, and enhanced monitoring systems to mitigate the health and environmental impacts of MPs in the MENA region.

## 1. Introduction

**Figure 1:**
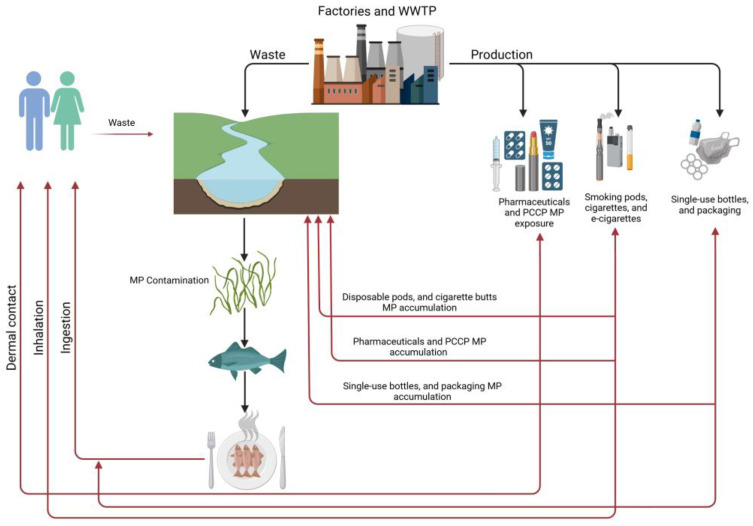
Illustrates sources of MP contamination.

The global production of non-biodegradable, oil-based synthetic plastics has reached over 400 million metric tons annually, with polyolefins—primarily polyethylene (PE) and polypropylene (PP)—dominating the market. PE accounts for approximately 100 million tons per year, while PP contributes an additional 80 million tons, driven by their versatility and low cost in applications ranging from packaging to healthcare [1,2]. However, the persistence of these materials in the environment has led to the unintended generation of microplastics (MPs) and nanoplastics (NPs), with an estimated 400,000 tons of MPs released into terrestrial and aquatic ecosystems annually [3]. MPs are categorized into two types based on their origin: primary MPs, which are intentionally manufactured for use in products such as cosmetics, textiles, and industrial abrasives, and secondary MPs, which result from the fragmentation of larger plastic debris due to abiotic stressors like UV radiation, mechanical abrasion, and chemical degradation [4]. In the United Nations Environment conference held in 2015, MP pollution was mentioned as the second largest global environmental problem, along with other ecological problems, including climate change and ozone depletion [5]. Mismanaged MPs (<5 mm) and NPs (<1 µm) are chemically stable and persist in the environment for extended periods. These particles are widely distributed through wind, water currents, and biotic carriers, infiltrating diverse ecosystems such as rivers, lakes, oceans, and forests. As shown in Figure 1, different factories and wastewater treatment plants generate waste, including MPs, which interferes with the ecosystem. MPs’ persistence leads to accumulation in these ecosystems, disrupting food webs, altering habitat structures, and impacting soil and aquatic health.

Different types of MPs are mentioned in Table 1, and the most common types are polyethylene (PE), polyvinyl chloride (PVC), poly (methyl methacrylate) (PMMA), polylactic acid (PA), polystyrene (PS), and polypropylene (PP) [6].

MPs have emerged as a significant concern for human health, with growing evidence suggesting their potential to induce gut microbiome disturbance, gastrointestinal disorders, cardiovascular diseases, and endocrine disruption. Studies indicate that MP accumulation can damage the blood–brain barrier (BBB), triggering inflammatory responses and oxidative stress in neural tissues, which may contribute to conditions such as Alzheimer’s and Parkinson’s diseases. Furthermore, the accumulation of MPs in the brain has been linked to the disruption of neuronal function, microglia activation, and the promotion of neurotoxicity, highlighting the urgent need for further research into their long-term neurological impacts [7].

**Table 1:** Explanation of different types of MPs, characteristics, applications, proposed effects on the environment, and volume produced.

Microplastic	Properties	Application	Environmental Impact	Volume Produced	References
Polyethylene (PE)	Low crystallinity, fair moisture, low density, and heat-sealable	Sterile blister packs, bottles, and sample containers	The most common form of waste in marine and coastal areas	32 million tons	[8,9]
Polyvinyl chloride (PVC)	Blister packs for pills, bottles, and ampoules through a thermoforming process	Good optical clarity and low cost	One of the most common wastes	36 million tons	[10]
Polymethyl methacrylate (PMMA)	High computability, functionality, low cost, and stability to ultraviolet light	Potential drug carrier	Carbon footprint	N/A	[11]
Polylactic acid (PLA)	Low cost and moderate mechanical property	Shrink wrap films	Eco-friendly	140,000 tons	[10,12]
Polystyrene (PS)	Hard, solid, and transparent	Disposable medical items and sterile plastic packaging	Water and air pollution	N/A	[13,14]
Polypropylene (PP)	Containers for extreme heat sterilization, thermoformed trays, and blow-molded bottles	Creep resistance, environmental stress cracking, and moisture barriers	Water and air pollution	45 million tons	[10]

## 2. Microplastics—A Global Problem of Our Time

There has been a dramatic escalation in global plastic production, surpassing 320 million tons annually, with over 40% dedicated to single-use applications. This surge has resulted in a substantial accumulation of plastic waste, a considerable portion of which has infiltrated marine ecosystems. The Mediterranean Sea, for instance, grapples with a significant plastic burden stemming from coastal and oceanic debris. MPs originate from diverse sources and exhibit complex environmental behavior influenced by inherent physicochemical properties and external factors. Their ubiquitous presence extends beyond marine environments, encompassing freshwater, atmospheric, and terrestrial systems. MPs have been reported in a wide range of consumer products, including seafood, sea salt, and even beverages.

Furthermore, their presence within the gastrointestinal tracts of marine organisms has been extensively documented. Concerns arise as these particles persist and accumulate within the food chain, with high potential toxicity and the ability to serve as vectors for pathogens and pollutants, causing harmful effects on different biological systems. MP ingestion has been reported in approximately 150 species across freshwater and marine ecosystems [15].

## 3. The Problem of MPs in the MENA Region

The MENA region contributes approximately 1.4% of the global plastic emissions into the oceans [16]. This region faces significant environmental challenges related to MP pollution, with the Maghreb’s coastal erosion rates influenced by MPs exceeding the global average by a factor of 10, reaching 7 mm per year [17]. Additionally, around 570,000 tons of plastic is discarded annually into the Mediterranean Sea, equivalent to 33,800 plastic bottles per minute. This pollution level leads to substantial economic losses for Mediterranean countries, with an estimated annual cost of nearly USD 770 million, impacting key industries such as tourism and fisheries [18]. Evidently, in the last 5 years, there have been 35,025, 29, and 21 published research studies on Pubmed, Scopus, and Web of Science addressing the problem of MPs in the MENA region, respectively (Table 2).

Regional studies have explored the sources and contributors to MP pollution across the MENA region. In Morocco, social behaviors such as the disposal of plastic waste—such as cups, containers, and straws—directly into seawater exacerbate MP contamination. Fishery-related activities involving nets, ropes, and packaging materials also contribute significantly to MP pollution [23]. In Iran, a study examined indoor classroom contamination across 50 schools, revealing approximately 80 to over 56,000 MPs per gram of dry dust, with significant concentrations in the center and southeast of the city [24]. In Egypt, research focused on the presence of MPs in marine organisms. A study found a substantial number of MPs in *Siganus rivulatus* fish, with over 1000 MPs per individual, attributed mainly to ingesting plastic particles broken down from larger plastic waste in polluted harbors. This herbivorous fish feeds on seaweed and inadvertently consumes MPs trapped within seaweed, compounding the issue [25]. In contrast, studies in Kuwait revealed comparatively lower MP pollution levels in coastal areas, with MPs detected in beach sediment, seawater, and fish, though still less than those reported in Qatar and Oman [20]. Similarly, in Lebanon and Tunisia, studies of oysters and mussels found an average of 7.2 MPs per individual in Lebanon and 7.7 MPs per individual in Tunisia, highlighting the pervasive nature of MP contamination in marine environments [21,22]. Table 2 shows the difference in the marine contamination in MENA region countries.

Consumer behavior and awareness regarding plastic use were also examined in Egypt, specifically in a community-based study conducted in Assiut. The findings revealed that only 24% of participants displayed adequate knowledge regarding single-use plastic products, while approximately 84% exhibited poor awareness of safe plastic types for food storage. These results align with previous research in Egypt, underscoring a widespread lack of public understanding of the safe use of plastics [5].

## 4. Sources and Pathways of MPs in the Biosphere

MPs pose environmental threats to marine biodiversity, ecosystems, animal health, livelihoods, fisheries, maritime transport, recreation, tourism, food safety, and the economy [26]. In the United Nations Environment conference held in 2015, MP pollution was mentioned as the second largest global environmental problem, along with other ecological problems, including climate change and ozone depletion [5]. MPs get discharged into the environment and enter the human body through different means. MPs can be transported from one environmental compartment to another; for example, plastics can be transported from the air compartment through the wind to the water compartment [16]. Due to the several available sources of MPs, it is not easy to attribute them to one source [27]. The hazards of MPs depend on their physical and chemical properties (size, shape, composition, and the environment in which they are found) [28]. MPs have been shown to decrease photosynthesis and microalgal growth, release harmful plastic additives, and induce pathogenic bacteria growth [29].

### 4.1. MPs in the Marine Environment and Freshwater

While many regions worldwide make substantial contributions to the seas, the MENA region records the highest footprint per capita of MP use and leakage into the region’s marine water [17]. It records the most MP-polluted seas and oceans, especially the Mediterranean Sea [17]. The Persian Gulf (a major fishing hotspot in Iran) showed MP pollution in fish tissue across all the examined fish types (*Platycephalus indicus*, *Alepes djedaba*, *Epinephelus coioides*, and *Sphyraena jello)* [30]. If the emergence of MPs in the ocean continues to be 5 to 13 million tons annually, it is expected that by 2050, the weight of discharged plastic will exceed the total weight of fish [27]. This shows the importance of investigating all the potential sources of MPs and how to eliminate them from marine water [27]. The high number of fibers is attributed to fishing gear (such as rope and net) [30]. A significant positive correlation (*p* < 0.01) between fish length and MP level has been observed. The high consumption of fish species containing MPs poses a health risk to consumers [31]. In Kuwait, high levels of MPs were detected in fish guts [32]. Consistently with Saudi Arabia, MPs seem abundant in fish tissues and various fish species’ digestive tracts (liver and muscles) [33].

Since MPs can quickly move among environmental media, high-density MPs can accumulate and settle in the bottom of the water environment, which makes it hard to determine the MP concentration in water environments [27]. The highest MP concentrations recorded in marine water (400 MPs/m^3^) and sediments (7960 MPs/kg) among the MENA region were in the Mediterranean Sea in Tunisia, as shown in Table 2 [16]. It is hard to measure the distribution and transfer of marine MPs due to their multifactorial nature (ocean hydrology, weather, MP physicochemical properties, and biological processes) [27]. Due to the inherent properties of MPs (including hydrophobicity and specific gravity), they are not evenly distributed in the water compartment. There are more MPs in seawater than on the water surface. Low-density MPs float on the water surface for 6–8 months, while spherical particles float for 10–15 years, then sink into the deep sea, increasing the concentrations of MPs in sediments [27]. Both raw and treated drinking water treatment plants (DWTPs) in Tehran, Iran, showed abundant MP levels, which were transmitted to humans through ingestion. The study concluded that DWTPs are not successful in eliminating MPs, and the study recommended increasing the depth of sand filtration, using finer particles, and disregarding plastic wipes for water transfer [34]. Freshwater in Iran has been reported to have high MP content, and it was noticed that the highest MPs were in areas close to roads, agricultural fields, and tourist centers (>90% of the MPs in water were fibrous MPs) [24]. As for plastic bottled water, in Saudi Arabia, it has been reported to include various types of MPs, and according to the recommended average water intake in hot weather (3.7 and 2.7 L per day for men and women, respectively), the average MP exposure is estimated to be 0.1–0.2 pcs/kg bw, which does not pose a concerning health risk [35].

Regional studies conducted in Egyptian cities and governorates showed a high content of MP particles in fish species. The Red Sea and the Mediterranean Sea are essential bodies of water for Egypt, supporting various human activities [36]. The research was focused on four fish markets located along these two seas. Fish samples were collected from Hurghada, Suez in the Red Sea, Alexandria, and Port Said in the Mediterranean Sea. A total of twelve fish species were investigated across these locations. MP examinations were conducted on six fish species intended for human consumption at four different sea sites in Egypt. The study found MPs of varying sizes, ranging across >5000 μmL, 500–1000 μm, and 500–1000, in the sampled fish [36]. Another study conducted in Egypt investigating commercial fish shared that the average MP particles found in fish samples were 91.8 ± 8.4% and 11.7 ± 9.5 MPs/fish and that the *Sparus aurata* species showed the most significant MP content of all the species that were analyzed, surpassed only by the *Boops boops* and *Siganus rivulatus* species [19,37]. This suggested that these fish species are most vulnerable to plastic waste in seawater.

On the Red Sea coast of Saudi Arabia, scientists researched 26 species of non-commercial and commercial fish for MP contamination. The study reported that 18 of the 26 species of fish were found to contain MP particles, and the average occurrence of marine plastic particles in these species was 14.4 ± 0.3%, indicating that ingesting MPs impacts a considerable fraction of the fish in the Red Sea. One of the species analyzed was *Parascolopsis eriomma*, which stood out as a distinctly noticeable example; three of the five samples investigated were found to have consumed MP particles. This indicated a 60% prevalence in the sample, emphasizing the species’ susceptibility to MP contamination [38].

Spiny oyster and anchovy samples from three locations were also examined to gauge the extent of MP waste in Lebanese seawater and biota [21]. The study discovered MP fibers, films, and fragments of MP particles in the aquatic organisms; fragments were more abundant than the others to a small degree. Anchovy and oyster samples gathered from Beirut coasts had the highest MP particle content of 2.9 ± 1.9 MPs and 8.3 ± 4.4 MPs per fish compared to the 2.3 ± 1.6 MPs per fish and 0.22 ± 0.13 per gram at the Tripoli site, respectively. Moreover, there was no correlation between the site where the anchovies were gathered and the amount of MP content found, which could be due to the frequent movement of the anchovies [21].

### 4.2. MP Contamination in Seafood

Plastic pollution is a problem worldwide. In developing countries, people rely on fish from aquaculture for protein. MPs in the environment can lead to fish misfeeding, which poses a risk to seafood consumption. People usually eat clean seafood, which removes the gastrointestinal (GI) tract. This can reduce exposure to MPs compared to eating whole fish. However, this is not the case for shellfish and certain small fish. MPs in seafood likely come from other sources, such as processing aids, water, air, or machinery. The amount of MPs or NPs may increase during the processing of seafood. The effects of cooking and baking on the content of plastics in seafood are not known [6].

An investigation of MPs in nine commercial fish species in the Arabian Gulf found them in 5.71% of the fish examined, with an average of 0.057 MPs per fish. The MPs mainly consisted of fishing threads and fragments. The study found no difference in the presence of MPs between fish from different habitats. Despite Saudi Arabia’s high level of industrialization, the prevalence of MPs in the Arabian Gulf is relatively low compared to other regions [38].

Another investigation of MP pollution in the marine environment in the Red Sea found MPs in sediment and fish samples. The MPs in the sediment samples ranged from 0 to 119 particles per kilogram of wet sediment. The MPs in the sediment were fragments, granules, foams, and fibers. MPs were found in 44% of the fish samples. The most common types of MPs found were polyethylene terephthalate and vinyl chloride–vinyl acetate copolymers in sediment samples and PS, PE, and polyester in fish samples. The study concluded that MP pollution is a serious threat to the marine environment of the Red Sea [39].

In Egypt, researchers found many MPs in *Siganus rivulatus* fish (more than 1000 MPs/individual). They believe this is because the fish were collected from a polluted harbor where plastic breaks down into smaller pieces that are easier for fish to eat. *Siganus rivulatus* is herbivorous fish that eats seaweed, and seaweed can trap MPs. This means that the fish may accidentally eat MPs when they eat seaweed [25]. A new method was used in Egypt to measure MP contamination in fish from the Eastern Harbor. The method combined visual counting, combustion analysis, and differential scanning calorimetry. Plastic particles were found in all fish samples, and seven different types of plastic were identified. The fish with the highest MPs were *Siganus rivulatus*, *Diplodus sargus*, and *Sardinella aurita*, while *Sphyraena viridensis* and *Atherina boyeri* had the lowest. The weight of MPs in the fish also varied, with *Siganus rivulatus* having the highest amount and *Terapon puta* having the lowest [25].

Many factors cause pollution along the Mediterranean and Red Sea coasts. These include the oil and gas industries, urban growth, tourism, fishing, shipping, resorts, and harbor activities [19,40]. Several studies have found similar levels of MP pollution in the eastern and western Mediterranean Seas.

### 4.3. Airborne MPs

Air pollution in the MENA region is second only to South Asia [17]. The average resident in the region breathes air 10 times more polluted than the permissible pollutant level [17]. Street dust in urban areas in Iran shows an abundance of MPs originating from industrial emissions, synthetic textiles, decomposing litter, waste disposal, and agricultural practices; this parallels previous studies confirming the abundance of MPs in the atmosphere [24].

Global warming can increase the levels of MPs by releasing plastic bound to ice in the Arctic Ocean [27]. Weather conditions have also been reported to influence the abundance of MPs (including rainfall, snow, typhoons, and other environmental factors) [27]. The highest MP concentrations in the Marmara Sea were recorded in the Autumn, and the lowest concentrations were recorded in the summer. In contrast, high MP concentrations in the southwest Baltic Sea were reported after rain and snowfall [27].

MP properties depend on the level of ventilation and airflow, which explains why higher MPs characterize indoor sites. Moreover, using synthetic materials in furniture and cleaning procedures induces MP production [29]. The primary source of MPs indoors is synthetic textiles due to the emergence of MPs from clothes and other fiber products during wearing, cleaning, and drying [29]. Also, the use of plastic packaging and 3D printers increases MP concentrations [29]. Road dust resulting from rubber tires in hot temperatures, soil alongside roads, and stormwater greatly contribute to MP pollution in the UAE, which is consistent with previous studies [41]. MPs contribute to climate change through their ability to release climate-altering gases during fragmentation [42]. Interactions across the environment among plastic waste, wind, ice, floods, waves, and sunlight can facilitate the degradation of large MP pieces into smaller debris [42]. Especially in humid weather, such as storms, abundant MPs originating from urban areas are deposited [42]. When MPs are abundant in water, they absorb sunlight, increase temperature and warm oceans, and alter water density, changing ocean currents and circulation patterns and the climate of the globe [42].

### 4.4. Influence of Geographical Location and Anthropogenic Activities (Industrialization and Urbanization) on MP Accumulation

Ocean currents, winds, and weather events influence the dispersion of MPs in seawater. The geographic variation related to coastal currents influences MP abundance in seawater [27]. The Mediterranean Sea is highly contaminated with MPs due to the naturally high population across coastal areas and major rivers (including the Nile and Rhone) and intense shipping activities; its semi-enclosed shape with a narrow strait compromises the ability of water to exchange with open access, which accordingly allows MPs to accumulate in surface water and sediments [43]. The Red Sea, in contrast, is more isolated, which gives it different dynamics of MP availability through maritime activities and shipping; however, in comparison to the Mediterranean Sea, the Red Sea has a lower intensity of MPs [44]. It has been observed that there are higher levels of MPs in urban areas than in rural areas; the difference is attributed to higher population growth and anthropogenic activities [29]. According to MP analysis, fiber-type MPs are the most dominant, followed by the fragment, film, and granule types [27]. Most of the fibers are derived from textiles and other anthropogenic sources, including the laundry of blue denim, and are transferred to waste water [27].

A correlation between intensive economic activities and compromised water quality has been found due to the release of high MP concentrations [27]. Additionally, the concentration of MPs is influenced by the surrounding environment, where studies show that worn tires and asphalt can flow into the water and increase the MPs emerging from cities [27]. The MENA region contributes 8.3% to the global mismanagement of plastic [16]. Exposure to airborne MPs through inhalation in industrial factories in Dubai was observed, primarily through processing synthetic fibrous materials (e.g., nylon, polyamide) or plastics, which compromises the health of the respiratory system of workers [34].

## 5. Sources and Pathways of MP Contamination in the Pharmaceutical Industry

### 5.1. MPs in the Pharmaceutical Industry

The pharmaceutical industry uses plastic and MPs in many stages, from manufacturing to packing. The role of MPs is vital; they can be used as a vector because MPs have unique properties. These properties include taste-masking and controlled release (CR) [45]. There are various mechanisms through which MPs exhibit CR properties.

Guaranteeing the safety and efficiency of drugs during storage, transportation, delivery, and use revolves around the usage of MPs. Since packing is necessary to enclose or protect the drugs to ensure that the medicines arrive to the patients safely, it should also provide safety, identification, and information about physical damage. Plastic has many advantages over the other materials used in pharmaceutical packing. It shows high versatility and unique properties that meet rigorous medical specifications and criteria [10]. Many types of MPs are used in the pharmaceutical industry, as illustrated in Table 3. Given that pharmaceutical packing is necessary for healthcare, there are three stages of packing: Primary packing defines the first layer that coats the active constituent. It includes strips, blisters, vials, and bottles. It protects the drug against contamination. It is followed by secondary packing, which facilitates the handling of the drug by using cartons and boxes. Then, the tertiary packing is intended for handling and transporting bulk. Safe shipment by containers and ships belongs to this category [9].

The pharmaceutical industry is one of the most regulated and monitored industrial segments. Yet, the usage of plastics and MPs is causing damage to the ecosystem, especially in developing countries in the MENA region. This is mainly driven by the regulations set by governments. Surprisingly, the Gulf region is estimated to produce 17 tons of PP pellets per hour. Subsequently, the annual equivalent emitted carbon dioxide is 27 tons [46]. However, this study used GaBi modeling software, version 4.2 to construct PP’s life cycle assessment (LCA). The same study also stated that the manufacturing of PP, especially in the terrestrial acidification (TA) phase, leads to soil infertility because of nitrogen- and sulfur-based acidic compounds such as ammonia and sulfuric acid.

A comprehensive study conducted on Ras Rakan Island, Qatar, found nine locations around the island containing MP particles ranging from 0 to 665 per kilogram. The authors expected this considerable amount of MPs to have originated from land-based sources in the Gulf in the past 20 years. PE and PP contributed the most to this MP pollution, approximately 54% and 24%, respectively. Many studies have shown that many sources contribute to MP contamination in the MENA region [47].

### 5.2. MPs in the Cosmeceutical Industry

It has been determined that MPs are a serious environmental pollutant, specifically when considering the cosmeceutical sector. The prevalence of MPs in cosmetics and personal care products (PCPs) has caused significant ecological and public health problems in the MENA region, where demand for these products has increased dramatically. After disposal, MPs remain in the environment, as they are employed as fillers and exfoliants in toothpaste, shower gels, and scrubs. The difficulties caused by the insufficient control of plastic materials in cosmetics result in the extensive dispersion of MPs into aquatic habitats [48]. Dermatologists are alarmed about MPs and NPs because of their widespread usage in cosmetic formulations and, consequently, the environment. Multiple investigations of these synthetic polymers have been conducted to determine the possible effects on dermatological homeostasis, inflammatory reactions, and disturbances to the physiological processes of the skin.

Some research has demonstrated that single-dose and long-term, chronic exposure to MPs/NPs results in premature aging, autophagic structures, and oxidative stress-mediated cell growth limitations [49]. Furthermore, a different study has shown that MPs raise reactive oxygen species (ROS) levels in rotifers in a size-dependent pattern. To lessen the oxidative stress brought on by MP exposure, a defense reaction is subsequently set off, which results in the activation of antioxidant-related enzymes such as superoxide dismutase, glutathione s-transferases, glutathione reductase, and glutathione peroxidase [50]. Additionally, comparable results have been shown in human and animal models, showing that plastic particles can cause cellular oxidative stress even at low concentrations and cause cytotoxicity in excess.

The environmental impact of MPs is particularly concerning in regions with scarce water resources, such as the MENA region. Conventional wastewater treatment processes do not effectively remove MPs, leading to their accumulation in marine environments.

The cosmeceutical industry is heading in the right direction to recognize the need for more sustainable practices as MPs’ health and environmental risks become more apparent. Researchers emphasize the importance of corporate responsibility in the sustainability assessment of significant cosmetics and personal care companies. Many companies in the MENA region are adopting sustainable development goals (SDGs), aligning their practices with global efforts to minimize environmental harm. For instance, some brands have started to explore alternatives to plastic microbeads, turning to natural ingredients like sugar, salt, and biodegradable beads. However, these efforts remain in the infant stages, and widespread change in this industry is still needed to mitigate MP pollution [25].

Developing biodegradable substitutes is a promising way to lessen the use of MPs in cosmeceuticals. The possibility of using seaweed-derived polysaccharides, like ulvan and alginate, as alternatives to plastic microbeads in exfoliating cosmetic products has been investigated [51]. These natural substances have two main advantages: they are safe for the environment and work well as exfoliants. Examining the application of bio-based polymers in cosmetics, which may provide sustainable substitutes for MPs, ref. [52] supports this strategy even further. Using such materials would promote the transition to eco-friendly formulations while keeping the interested parties of the industry interested.

Many databases and initiatives aim to mitigate the risks associated with MPs in cosmetics. The Beat the Microbead Initiative is a global campaign advocating for eliminating plastic microbeads in cosmetics and personal care products. Remarkably, this initiative has developed a public database of products containing MPs, and it works closely with governments and industries to push for global bans on MPs in cosmetics. Similarly, the Cosmetics Ingredient Review (CIR) database was developed to evaluate the safety of ingredients used in cosmetics, including those related to plastic microbeads. This database helps companies identify safer and more sustainable alternatives to MPs, supporting efforts to reduce their environmental impact [53]. These initiatives and databases play a crucial role in promoting sustainable practices in the cosmeceutical industry and reducing the environmental and health risks caused by MPs.

The WHO has expressed concerns over MPs’ potential human health impacts, especially in drinking water. The organization has called for further research into the effects of MPs on human health and recommended regulatory frameworks that prevent environmental contamination. However, direct WHO regulations specific to MPs in cosmetics are still under development [54]. The (International Organization for Standardization) ISO should play a crucial role in establishing standards related to environmental management and plastic pollution [53]. Although the ISO does not have specific standards for MPs in cosmetics, ISO 14001 and related environmental management standards can guide industries toward reducing plastic waste, including MPs, through sustainable practices [53]. These standards encourage companies to implement systems that mitigate pollution and environmental risks, supporting the reduction in MP contamination in water and soil [53].

The WHO and ISO actively contribute to broader discussions and frameworks that address MPs’ environmental and health impacts. However, their regulations are more focused on plastic pollution than specific cosmetic applications or the risks posed to consumers.

Nearly all of L’Oreal’s personal care and cosmetic products previously contained microbeads, particularly in exfoliating scrubs and cleansers. The company has pledged to phase out MPs and is working on more sustainable product formulations. Similarly, Procter & Gamble, a global leader in the personal care industry, has also been known to use MPs in various products. Beat the Microbead (2023) identified P&G as one of the companies that utilized MPs in rinse-off products like body washes and toothpaste. Subsequently, the company is now focusing on finding alternatives to these plastic particles. Unilever, another global player in the PCCP sector, has also been known for using MPs in products such as exfoliating cleansers. They prioritized sustainability and have been actively working to replace plastic microbeads with environmentally friendly alternatives as part of their commitment to sustainable practices [53].

## 6. The Impact of MPs on Human Health

MPs can enter the human body primarily through dermal contact, inhalation, and ingesting food additives and water. Dermal contact allows MPs to enter the human body; however, dermal absorption is unlikely to happen through the skin’s stratum corneum. Moreover, human inhalation of MPs through indoor air exposure is higher than through outdoor air exposure [55]. This is alarming because people usually spend 70 to 90% of their time indoors [29]. The human body has a natural self-defense mechanism that prevents the deposition of MPs (including sneezing, coughing, mucociliary escalator, and lymphatic transport), and chronic exposure may cause the development of asthma, as MPs could reach alveoli [29]. However, if we estimate that the average adult inhales 1 MP/m^3^ per breath per day, about 6351 MPs would be inhaled yearly, which is known to impose long-term human health risks [29].

Importantly, the ingestion of MPs is considered to be the highest threat because it allows MPs to accumulate in the human body within different organs. For example, MPs usually constitute about 4% of the total weight of food additives. This means that a small amount of the MPs we ingest are additives. One example of an additive is bisphenol-A (BPA). A mussel containing 7 μg of MPs would also contain about 0.28 μg of BPA. In the worst-case scenario, we could assume that all of the BPA in the MPs would be released. According to the European Food and Safety Authority (EFSA), the average daily exposure to BPA from all sources is about 0.19–0.20 μg per kilogram of body weight per day. Therefore, a 70 kg adult would ingest about 14 μg of BPA per day. This means that the BPA from MPs in mussels would only contribute a small amount, about 2%, to the total daily exposure to BPA. Exposure to other additives containing MPs is also expected to be low [6]. Another study analyzed the amount of MPs in salt brands available in Lebanese markets, highlighting that most salt samples contained MP particles.

**Figure 2:**
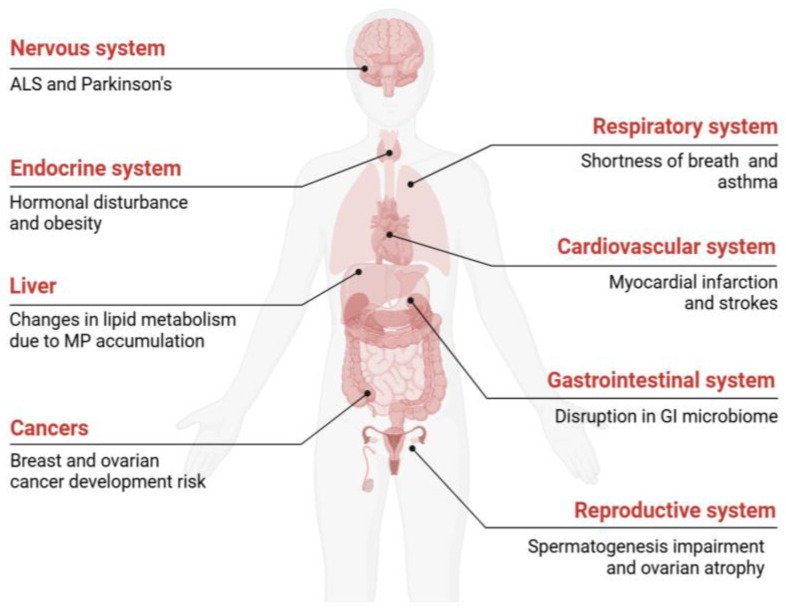
The toxic impact of MPs on human health.

The sample analysis showed that 84.6% of packaged salt items and coarse salt contained high concentrations of MPs [56]. Lebanese individuals consume approximately 2372 MPs per capita^−1^ year^−1^ through salt ingestion [56]. A recent study which estimated the average intake of MPs through drinking water by adults in Saudi Arabia found that 57% of the bottled and tap water samples had particles ranging from 25 to 500 μm [35]. Accurate data on the daily MP intake in the human diet are limited, even more so in the Middle East and North African region, and research outside the region shows that commonly consumed items containing MP particles threaten the human body, as shown in Figure 2.

### 6.1. Impact on the Gastrointestinal System

MPs are linked to digestive disorders that disrupt the gut microbiome. The four dominant phyla in the human gut are *Proteobacteria*, *Actinobacteria*, *Firmicutes*, and *Bacteroidetes*. Studies show that *Bacteroidetes*, which have an anti-inflammatory effect on the guts, constantly decrease in mice and zebrafish when exposed to MPs [57]. Moreover, fecal MPs increase even after the cessation of exposure. For example, when MP exposure to mice is cut off, the abundance of the key phyla *Bacteroidetes* and *Firmicutes* is dramatically reduced [57].

Furthermore, the effects of MPs were tested on strains and examined using an in vitro culture method; the results showed that PS MPs significantly inhibited the growth of *Lactobacillus*. It was concluded that the digestive system cannot break down MPs, which may lead to an imbalance in the intestinal microbiota [58]. Scientists have explored MPs’ potential digestive health effects using harmonized static and dynamic gastrointestinal models. This study reveals the significant finding that MPs can alter the composition of the human colonic microbial community. This alteration may occur after MPs adhere to colonic microbiota, further promoting biofilm formation [59].

### 6.2. Impact on the Cardiovascular System

A team of researchers is working on a prospective, multicentered, observational study to determine if MPs and NPs are detectable in atherosclerotic plaque and to assess their association with cardiovascular disease. Researchers have analyzed surgically excised carotid artery plaque using pyrolysis–gas chromatography–mass spectrometry, stable isotope analysis, and electron microscopy. They then evaluated whether the presence of MPs and NPs was linked to a composite endpoint of myocardial infarction, stroke, or death from any cause [60]. The primary endpoint is a composite of nonfatal myocardial infarction, nonfatal stroke, or death from any cause among patients with MP- and NP-containing plaque compared to those without. Secondary endpoints include the levels of tissue biomarkers such as interleukin-18, interleukin-1β, tumor necrosis factor α (TNF-α), interleukin-6, CD68, CD3, and collagen in patients with MPs and NPs versus those without. A total of 312 patients undergoing carotid endarterectomy were screened. Among these patients, eight experienced a stroke or died before being discharged from the hospital; in one hundred and fifty patients (58.4%), detectable amounts of PE were found in excised carotid plaque, and thirty-one of these patients (12.1%) also had measurable amounts of PVC in their carotid plaque. Various studies in mice and rats have demonstrated a widespread distribution of MPs and NPs following inhalation and ingestion and consistent accumulation in highly vascularized organs and the heart [60].

### 6.3. Impact on the Endocrine System and Hormones

MPs are linked to endocrine disturbance and body mass index increment. A review of human exposure to MPs and obesogens hypothesized that the worldwide obesity crisis may be linked to exposure to obesogens and plastic additives [61]. Obesogens are chemicals that cause the increased accumulation of white adipose tissue after exposure in living organisms. MPs are found to impact adipocyte differentiation after accumulating in the liver and kidneys, leading to changes in energy balance and lipid metabolism [62]. Researchers have reported a decrease in triglycerides and total cholesterol levels in mouse liver following exposure to 5 and 20 μm PS MPs and confirm that MPs induce lipid metabolism imbalance and metabolic alterations, including decreases in ATP production and lipid metabolism. Changes in liver lipid profiles and lipid metabolism following exposure to PS MPs have been documented in several other in vivo mouse exposure studies and in vitro human cell bioassays [62].

### 6.4. Impact on the Reproductive System

A study conducted on forty-eight female rats has shown that the co-exposure of PS and di-ethylhexyl phthalate (DEHP) for forty-two days leads to follicular structural abnormalities such as flocculent oocyte nuclear chromatin and granulosa cell detachment and hemorrhage. Additionally, the incidence of secondary and atretic follicles was slightly higher in the co-exposure groups than in the control groups. Ovarian fibrosis was also noticed in the co-exposure groups due to collagen deposition. Transforming growth factor β1 (TGF β1) plays a vital role in fibrosis and ovarian atrophy. It has been elucidated that the TGF β1/Smad3 pathway is significantly higher when exposed to PS and DEHP [63]. Another study stated that maternal PS exposure leads to decreased sperm count and reduced testicle weight, causing spermatogenesis impairment [64]. Thus, the observed reproductive abnormalities in rats and mice due to MP exposure raise concerns about the potential complications in human health.

### 6.5. MPs’ Impact on Cancer Risk

A group of researchers has assessed the effect of PP on human breast cancer cells. Since breast cancer involves chronic inflammation, it was essential to measure the interleukin levels. It was found that IL-6 was elevated in the PP-exposed cell lines. Additionally, there was a significant overexpression of TMBIM6, AP2M1, and PTP4TA after incubation for only 12 h [65]. Furthermore, peer-reviewed published research has found that workers who are exposed to PVC are more likely to develop different types of cancers, such as angiosarcomas and hepatocellular carcinomas [66]. A study addressing the impact of MPs in the cancer microenvironment has stated that the microenvironment is enriched when exposed to PS. Moreover, there is an elevated expression in the epithelial ovarian gene. It was also observed that there is a mitotic count increment that is 2.8 times higher than the control group. PS also has a negative effect on cell viability, decreasing to 36.1% and accelerating epithelial ovarian tumors [67].

### 6.6. MPs’ Impact on Nervous System

**Figure 3:**
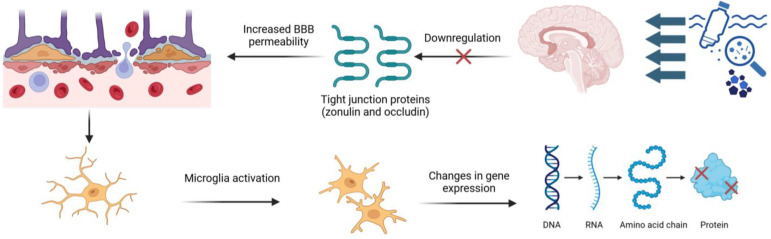
Toxic effects of MPs on nervous system.

Alterations in the gut microbiome and hormonal disturbance are the main factors behind blood–brain barrier (BBB) damage. The BBB is a critical barrier that protects the brain from peripheral toxins and pathogens. As shown in Figure 3, MPs accumulate in brain tissue because they downregulate tight junction proteins, such as zonulin and occludin, leading to increased BBB permeability [68]. This effect has been observed in key brain regions, including the cortex, hypothalamus, and hippocampus, making the brain more vulnerable to systemic inflammation and circulating toxins. In vivo studies, including those using chicken models, have revealed structural damage in the brain, such as intracerebral hemorrhage, associated with MP exposure. This structural damage is often accompanied by mitochondrial dysfunction, a critical factor in maintaining cellular energy homeostasis. Mitochondrial damage increases the production of ROS, exacerbating oxidative stress and amplifying neuronal damage [69,70]. Furthermore, PS has been internalized by human cerebral microvascular endothelial cells (hCMEC/D3), which are widely used as models to study human BBB function. These cells exhibit altered surface properties and impaired functionality following PS exposure, validating the neurotoxic potential of MPs in human systems [69].

Studies in animal models, particularly mice, have demonstrated that MPs contribute to neuroinflammatory processes and are associated with the onset and progression of various neurodegenerative diseases. Among these particles, polystyrene (PS), a common type of MP, has garnered attention due to its pronounced neurotoxic effects [68]. Research indicates that PS exposure predominantly activates microglial cells, the primary immune cells in the central nervous system (CNS), rather than directly affecting neurons or astrocytes. Transcriptomic analyses have shown that PS induces significant changes in gene expression associated with microglial activation, leading to the polarization of these cells. Activated microglia express M1 and M2 phenotypes associated with pro-inflammatory and anti-inflammatory states. However, the pro-inflammatory response is particularly pronounced in the context of PS exposure. This response is characterized by releasing cytokines such as TNF-α, various interleukins, and chemokines, contributing to a neuroinflammatory milieu that exacerbates neuronal damage [71].

In vitro experiments further elucidate the impact of PS on hippocampal neurons. When microglia are exposed to PS, neuronal function is significantly suppressed, highlighting the indirect but profound neurotoxic effects mediated by microglial activation. Beyond cellular and molecular effects, MPs are shown to induce behavioral deficits in animal models, including impairments in memory, learning, and motor function. These behavioral changes are linked to disruptions in cholinergic signaling, a critical neurotransmitter pathway for cognitive processes, and are accompanied by increased oxidative stress, a key driver of cellular damage [72].

PS interacts with critical neuronal proteins, such as Tar-DNA binding protein 43 (TDP-43), at the molecular level. TDP-43 is a key protein in amyotrophic lateral sclerosis (ALS) and other neurodegenerative diseases. PS induces the hyperphosphorylation and aggregation of TDP-43, leading to protein misfolding and toxicity. Additionally, MPs promote the aggregation of alpha-synuclein, a hallmark of Parkinson’s disease and dementia. The aggregation of these proteins disrupts neuronal function, accelerates disease progression, and contributes to cognitive decline [71]. Interestingly, maternal exposure to MPs is identified as a risk factor for neurological and cognitive impairments in offspring. Behavioral studies, including the Y-maze and novel object recognition tests, demonstrate deficits in memory and exploratory behavior in the progeny of exposed animals. These findings underscore the transgenerational effects of MP exposure on the CNS [73].

## 7. Policies and Regulations

### 7.1. Global-Scale Efforts Addressing MPs

Due to the tremendous increase in MP pollution, many policies were developed, with identifiable flaws that challenge the effectiveness of implementation [26]. Moreover, the slow implementation does not parallel the radical increase in the addressed problems affecting the globe differently [26]. Policies addressing MPs have primarily neglected the effect of agricultural sewage and plastic-coated fertilizers [26]. The UN has provided international communities with data explaining how MPs pollute seawater and, accordingly, human health, along with guidance on how countries can control the effect [26]. According to the 2021 SDGs Report, the UN has identified marine plastics and MPs in 13 of its 17 SDGs [26]. However, only one indicator out of the two hundred and forty-seven indicators of the SDGs addresses MP pollution. An internationally accepted index does not yet exist, which adds to the challenges of on-ground policy implementation, reporting, and monitoring [26].

In 2019, the WHO called to examine the prevalence of MPs and their influence on human health; it called for reducing MPs, conducting extensive research to analyze the accurate effect on human health, and developing and standardizing the methods of measuring MPs in water. In addition, water companies should be urged to prioritize removing MPs from drinking water since effective wastewater treatment against chemicals and pathogens will remove 90% of the MP content [26]. Most importantly, the Committee on Food Additives and Contaminants (CODEX) report has illustrated the importance of identifying standard reference material to identify, quantify, and examine MP contamination in food [74].

### 7.2. Policies in the MENA Region

According to the World Bank’s Regional Director for Sustainable Development for the MENA region, marine plastic is an environmental challenge that no single country can address on its own; this is why regional coordination is essential [75]. In March 2021, an event was held across several countries in the MENA region (including Egypt, Lebanon, and Tunisia) in addition to other countries (including Canada and Côte d’Ivoire) to discuss marine pollution [18]. Three main policies were agreed on: (1) to add incentives and investment plans that focus on reducing plastic waste, converting MPs into valuable resources and building a green economy; (2) to set global targets and apply international commitment; and (3) the collaboration of public and private sectors to produce harmonized frameworks, adopt standard regulations, and ensure international commitments [18].

However, in 2019 in Egypt, as part of Egypt’s efforts towards achieving the SDGs of 2030, the Ministry of Environment announced its work on formulating a strategy to mitigate SPUB production through providing compensatory incentives for single-use plastic producers, in addition to providing them with EUR 6 million in subsidies to build new production lines of biodegradable bags. Moreover, the European Union has granted the ministry USD 2 million to fund scientific research for the initiative [76], as well as increasing the recycling rate to 25% by 2030 [77].

The multifaceted problem of MPs affecting the region calls for collaborative work among stakeholders in the private and public sectors [17]. Developing clearly defined policies allows the provision of constant monitoring and reporting systems and raises public awareness to facilitate behavioral change and environmental data collection [17]. For example, the UAE offers real-time air quality data, with guidance on tackling and reducing air pollution [17]. Also, the Qualit’Air program in Morocco represents an example of public awareness about air pollution through an online platform [17,26].

Even though plastic bags are a significant concern in most African countries, the law banning plastic bags is challenging to implement due to the resistance of major beneficiaries from the industry, poor enforcement, and lack of alternatives [26]. In Africa, 34 out of 54 countries have set policies that ban plastics. However, 16 of the 34 countries have imposed laws that ban plastic bags without prior guidelines to enable implementation [26]. Even though the MENA region does not lack policies to manage MP pollution, the lack of adequate planning and appropriate disposal and collection services, the use of inappropriate technology, inadequate financing, the limited availability of a trained and qualified workforce, and fast population growth collectively remain the main factors in the slow improvement in waste management [77].

## 8. Mitigation Strategies and Recommendations

### Public Health Interventions

MPs have become a crucial component in the modern world [42]. Their large-scale use has impacted our oceans, air, soil, and human health, which calls for tackling this issue considering the One Health Approach [42]. This calls for the collaborative commitment of consumers, government, and companies [44]. Eliminating plastic use is almost impossible, and some of the recommended solutions can be recycling, circular economy, waste management, and consumer awareness, which can reduce the burden of MP pollution [44]. It is essential to engage the plastic industries and ask them to develop solutions to mitigate MPs’ inadequate disposal [17]. Moreover, this increases the prices of fossil fuels used to produce plastic [17].

The obstacles to achieving the set policies must be analyzed and mitigated, and effective strategic planning must be adopted to meet country-based goals in the MENA region [77]. Developing governorate-based policies that protect against agricultural contamination is essential for food quality assurance [77].

It is urgent to develop policies that ensure food safety to prevent human exposure to MPs [77]. Effective policy implementation is necessary, but guidelines must be offered to facilitate the application process [77]. Global collaboration is needed to implement practical solutions to the MP crisis [28]. However, the world is producing more bioplastics, reaching around 2.4 million tons annually [78]. Bioplastics are eco-friendly and easily destroyed in a short time. Despite this, we still need to study the different types of bioplastics. The transition to destructible plastics like Poly(butylene succinate-co-adipate) (PBSA) offers potential benefits for mitigating plastic pollution but introduces significant ecological risks. These include increased fungal plant pathogens from biodegradation processes, alterations in soil microbial communities, and the potential formation of persistent MPs, disrupting nutrient cycling, soil health, and forest ecosystems. Additionally, incomplete degradation in specific environments may lead to long-term pollution, necessitating careful management and ongoing research to balance the environmental benefits with potential ecological impacts [79].

## 9. Conclusions and Future Directions

In conclusion, MP contamination in food and drugs poses significant health risks, including respiratory, gastrointestinal, cardiovascular, endocrine, neurodegenerative, reproductive, and neuroinflammatory disorders. Moreover, it exacerbates inflammation, leading to different types of cancer. The presence of MPs in the MENA region is dramatically increasing because of urbanization, plastic packaging practices, and inadequate regulatory frameworks. Despite various studies, there is a notable gap in region-specific research on long-term health impacts, and few effective policies are in place. Policymakers must develop stricter plastic-use regulations, promote sustainable alternatives, and implement public health campaigns to raise awareness and reduce exposure in this region.

## Figures and Tables

**Table 2:** Comparison between different countries in the MENA region regarding marine contamination.

Country	Area	Amount	Polymer	Physical Form	Reference
Egypt	Mediterranean Sea	7525 MPs/individual	PP and PE	Filaments and foams	[19]
Iran	Arab Gulf	0.71 MPs/m^3^	PP	Granules and fibers	[16]
Kuwait	Mediterranean Sea	1–5 MPs/individual	PP	Filament	[20]
Lebanon	Mediterranean Sea	2433 ± 2000 MPs/kg	PP	Fragments	[21]
Tunisia	Mediterranean Sea	400 ± 200 MPs/kg	PP and PE	Fibers	[22]

**Table 3:** The different mechanisms for MPs to exhibit CR properties [45].

Mechanism	MP Type
Diffusion through a polymer matrix or polymer membrane	Ethyl cellulose, carbomer polymers, polyvinyl acetate phthalate, and polyethylene vinyl acetate.
Ion exchange through cross-linked resins (ion exchange resins)	MPs containing calcium/sodium/hydrogen polystyrene sulfonate and methacrylic.
Osmotic control via semi-permeable membrane	Non-degradable polymers and water soluble.
